# The impact of a multimodal professional network on developing social capital and research capacity of faculty at historically black colleges and universities

**DOI:** 10.1007/s10639-022-11464-z

**Published:** 2022-12-01

**Authors:** Jillian Ives, Brian Drayton, Kathryn Hobbs, Joni Falk

**Affiliations:** TERC, 2067 Massachusetts Ave, Cambridge, MA 02140 USA

**Keywords:** Social capital, Professional networks, Faculty development, Minority serving institutions

## Abstract

This qualitative case study examined how a multimodal professional network environment (STEM for all Video Showcase) affected five STEM educational researchers’ capacity to engage in grant funded research at U.S. Historically Black Colleges and Universities (HBCUs). Guided by the social capital and professional network literature as a conceptual framework, we analyzed data from surveys, interviews, and online discussion posts. We aimed to understand HBCU-based researchers’ supports and barriers in writing and/or conducting grant funded research in STEM education, and ways in which the multimodal professional network experience supported their research and professional networking, if at all. We found that organizational structures shaped participants’ social capital as well as their grant funded research activities. Further, participating in a multimodal professional network enabled participants to further develop their research capacity and to also expand their collegial networks. We offer recommendations for institutions to support the research endeavors of their faculty and suggest ways in which organizations using or developing professional multimodal networks can enhance faculty research development.

## Introduction

The imperative to mount a significant research program as a way to enhance prestige has increasingly affected institutions of higher education whose historical mandate has not solely focused on scholarly productivity (Johnson & Harvey, 2002). This push for prestige influences the work of all faculty who feel the pressure to obtain revenue-generation grants, including those at Historically Black Colleges and Universities (HBCUs; Beach et al., 2008; Betsey, 2007). Yet there is inequity among institutional types when it comes to the resources and structural supports to enhance faculty grant funded research capacity. HBCUs receive a lower share of state and federal funds for research and development compared to predominantly white institutions (Boland & Gasman, 2014; Pece, 2018, 2019; Sav, 2010). This lack of funds often results in inadequate laboratory spaces (Qazi & Escobar, 2019) and understaffed sponsored programs offices to assist faculty with grant proposal writing and management (Matthews & Coleman, 2011).

The majority of HBCUs are primarily teaching institutions (Betsey, 2007; Ellis, 2012). As institutional mission shapes faculty work (Twombly & Townsend, 2008), faculty at these institutions have high teaching and service responsibilities (Ellis, 2012). While many faculty at HBCUs value this work (Ellis, 2012; Wright, 2009), balancing a high teaching and service load leaves them little time for research, including seeking and managing grants (Beach et al., 2008; Ellis, 2012). Furthermore, faculty can experience challenges in expanding their professional network, as scholars have found collaboration and mentorship can be lacking in HBCU research culture (Qazi & Escobar, 2019).

Scholars have shown that professional networks play a mediating role in faculty success (Warner et al., 2016; Zambrana et al., 2015), especially for collaboration outside of one’s own institution (Bankart, 2012; Hitchcock et al., 1995). Faculty collaboration across institutions is becoming increasingly necessary to be competitive for federal grants, as evidenced in calls for proposals (Dopke & Crawley, 2013; Eddy, 2010; Lee & Bozeman, 2005). Furthermore, professional networks can enable faculty to share and disseminate their work in progress, expand their knowledge (Carter-Thomas & Rowley-Jolivet, 2010; Rowley-Jolivet, 1999), and establish connections for future collaborations (Falk et al., 2019).

In this qualitative case study, we examined how a multimodal professional network—the STEM for All Video Showcase—influences U.S. HBCU-based researchers’ capacity to engage in grant funded research through the lens of social capital. We found that organizational habitus and field shaped the study participants’ social capital and grant funded experiences, and the professional network provided participants an opportunity to develop their research and expand their network. We conclude with implications for practice, policy, and future research on expanding HBCU-based researchers’ grant funded research.

## Research questions

1. What do researchers at HBCUs experience as supporting or hindering the development of their capacity to write and/or participate in grant funded research in STEM education?
2. How, if at all, does the STEM for All Video Showcase support the capacity of HBCU-based researchers to write and/or participate in grant funded research in STEM education?
  a How, if at all, does the Video Showcase increase the professional networks of faculty at HBCUs?

## Theoretical framework

We ground this study in Bourdieu’s (1986) sociological theory of practice as well as research on professional faculty network literature. Bourdieu was concerned with how society reproduces the social order through the inequitable transmission of capital and resources. In this article, we take an agentic view of faculty research—considering actions that educational researchers take to expand their network and engage in grant funded research within larger structures and contexts. In this section, we discuss the meaning of Bourdieu’s concepts of *field*, *habitus*, and *capital*, and how they relate to educational researchers in this study, and then expand upon the contemporary professional network literature.

The *field* is the broader social world in which individuals are situated, within a milieu of relations between individuals and institutions (Bourdieu, 1983; Grossman, 2013). Scholars have examined how the field affects faculty work, for example, ways in which faculty use the institutional field to make sense of their university’s aspirations for prestige as a research institution (Gonzales, 2013, 2014). We conceptualized the field as higher education with its federal funding schemas and structures. This field situates the individual researcher within pre-existing conditions that support or hinder their capacity for grant funded research. For example, we discussed previously the push for multi-institution collaboration in federal funding calls for proposals (Dopke & Crawley, 2013; Eddy, 2010), which leaves researchers reliant on their ability to create connections with other researchers.

Situated within the field is the *habitus*—one’s personal history and how one is socialized to see the world. As formulated by Bourdieu, one’s habitus influences attitudes and perceptions, and subsequent behaviors within a field (Bourdieu & Passeron, 1977; Bourdieu & Wacquant, 1992). Scholars have extended Bourdieu’s concept of personal habitus to organizations (e.g., McDonough, 1997), examining how organizational “practices, beliefs, and rules not only provide meaning but also structure social interaction” (Horvat & Antonio, 1999, p. 320). Within this study, we conceive organizational habitus as institutional supports and barriers that shape researchers’ grant funded experiences and behaviors. For example, HBCUs are often sought out as partners in multi-institution grant proposals (whether for perceived diversity or substantive collaboration; Warren et al., 2019). Yet many HBCUs lack financial offices or staffing to manage large grants (Matthews & Coleman, 2011), and so they may be relegated to a sub-award position rather serving as lead.

Habitus provides *capital*, which is assigned value within a field. Bourdieu conceptualized multiple types of capital, but for the purposes of this study we focus specifically on *social capital*. Social capital provides people a durable network of relationships which can be mobilized for resources and benefits (Bourdieu, 1986; Grossman, 2013). Everyone’s habitus provides capital, but within a specific field certain types of capital are given more value than others, thus reinforcing Bourdieu’s (1986) notion that capital reproduces the social order. More recently, scholars have also examined how socio-demographic variables such as race, class, and gender influence capital (e.g., Grossman, 2013; Parks-Yancy et al., 2008).

While in this paper we focus on the interaction of organizational context and professional networks with research capacity, we acknowledge that researchers’ race and ethnicity are inextricably intertwined with their grant funded experiences. As approximately two-thirds of HBCU faculty hold racially minoritized identities (Gasman, 2013), scholars have examined how access to mentoring and professional networks is racialized (Zambrana et al., 2015). For example, racially minoritized faculty face review process bias in funding (Gallo et al., 2022; Pece, 2018, 2019; Stuart, 2012), inequitable distribution of service (e.g., committee work) which limits research time (Domingo et al., 2020), inequitable access to culturally relevant mentorship (Mayo & Chhuon, 2014; Serrano, 2013), and limited co-author networks (Warner et al., 2016). While much of this scholarship is focused on racially minoritized faculty experiences at predominantly white institutions, these challenges and barriers also exist for racially minoritized faculty working at minority-serving institutions (MSIs; Ellis, 2012; Martinez et al., 2017).

The social capital literature is closely related to the professional network literature because social capital is gained from the ties between people in a network (Coleman, 1988). In this sense, professional networks can foster relationships outside one’s immediate community that give people access to social capital (Putnam, 2000). Such networks are open, large associations of people with some common interest but have weaker ties than in one’s immediate community (Field, 2008; Schuller & Theisens, 2010). As they are less dense and more heterogeneous, networks tend to allow people to share ideas and knowledge across communities (Schuller & Theisens, 2010), and find partners for future collaborations (Falk et al., 2019). In this study, we conceptualize the STEM for All Video Showcase as a professional network that creates ties in which social capital may reside for STEM educational researchers.

## Context of this study

The context of this study is an online, multimodal professional network consisting of the STEM for All Video Showcase (Video Showcase for short) and the related STEM for All Multiplex site. The Video Showcase (https://stemforall2021.videohall.com/) is an annual eight-day interactive event where over 1,000 researchers create, share, and discuss three-minute videos about their federally funded programs. Videos presented in 2021 have been viewed (to date) over 50,000 times by over 100,000 unique visitors across the globe. During the one-week event over 7,000 discussion posts were exchanged, as facilitators, researchers, educators, and other stakeholders offered comments, resources, and feedback to presenters. Refer to Falk et al. (2019) for an in-depth description of the platform and event.

The STEM for All Multiplex site (https://multiplex.videohall.com/) aggregates presentations from the Video Showcase over multiple years and now houses over 1,600 easily searchable, short videos of federally funded work to improve STEM in pre-K through graduate education. It also engages the community of researchers throughout the year by providing month-long themes, drawn from the vast collection of video presentations. Each theme includes an interactive webinar, a curated playlist of videos, online discussions, resources, a blog, and a synthesis brief.

Both platforms facilitate interactions and connectivity between researchers to improve STEM teaching and learning. Researchers who are working on federally funded grants learn of each other’s work in progress, offer feedback to one another, and disseminate their projects broadly to other researchers, educators, and the public at large, nationally and internationally. We say that the Video Showcase is a multimodal environment, because it affords participants with several interacting resources for learning and meaning-making (Adami, 2016; Pauwels, 2012). The two modalities most important for this study are the video presentations and the discussions that accompany each presentation. The capacity of video to transmit visual narrative, voice, text, data displays, and physical interactions such as gesture and physical juxtaposition in a conversation, make possible a thick presentation of ideas, contexts, and personalities. The discussions, intrinsically interactive, make accessible a wide range of additional transactions, by which participants can negotiate future professional relationships (Falk et al., 2019).

## Methodology

We employed a case study methodology to examine faculty experiences with grant funded research at HBCUs. This methodology was well suited to answer the research questions, as cases typically revolve around a “how” question on a contemporary, real-life phenomenon (Yin, 2003). Case studies also suggest that there is something significant that can be learned from the case that can speak to the larger phenomenon (Walton, 1992). This case study revolves around the question of how the Video Showcase supports research capacity. The case is bound by the individual participants and their experiences and speaks to the larger phenomenon of how multimodal professional networks can support research capacity at HBCUs.

### Sampling

We used purposeful sampling to recruit study participants (Patton, 2002), which is appropriate for investigations aiming for deeper understanding of specific populations (Ishak & Bakar, 2014). We narrowed down our sample using four criteria. First, we selected participants who were working at an HBCU at the time of their Video Showcase presentation. Second, we selected participants who were serving in a faculty, researcher, or administrative position on a federally funded study. Thus, we refer to our population as researchers rather than faculty. We chose this criterion because an important focus of our theoretical framework was to examine the impact the Video Showcase had on participants’ future research, including collaborations and connections. Our last two criteria for participant selection focused on their Video Showcase roles and interactions. We selected presenters and/or co-presenters from the 2020 or 2021 Video Showcases so participants could recall the recent experience. Lastly, we selected participants who had a moderate level of engagement in the Video Showcase. We defined a moderate level of engagement as at least 10 discussion posts on their own video and at least one comments on others’ videos, as the median number of posts was 21. 

While it is important to explore why some participants did not fully engage in the Video Showcase discussions, this is beyond the scope of this paper. Nine Video Showcase participants met all the above criteria, of which five agreed to participate in the study. Our research design, permissions, and instruments were reviewed and approved by the Institutional Review Board.

### Participants

The five study participants were STEM educational researchers in higher education; all were women, although gender was not a sampling criterion. Refer to Table 1 for the characteristics of study participants. All the participants had a video in the 2021 Video Showcase (two of whom had two videos), and one also had a video in the 2020 Video Showcase (participating two consecutive years).

**Table 1:** Participant characteristics

	n = 5
Institution Type	
Public HBCU	3
Private HBCU	2
Discipline	
STEM	2
Social Science	2
Education	1
Position	
Faculty	2
Administrator	2
Researcher	1
Race/Ethnicity	
Black	4
White Non-Hispanic	1

### Data sources

Case studies typically use multiple sources of data (Yin, 2003). For this case study, we drew on: surveys, interviews, and online discussion posts. The pre-interview survey included a demographic questionnaire and questions on career stage and work history. These questions allowed us to verify that participants met the sampling criteria, as well as to develop a case profile. After completing the survey, each participant completed one semi-structured interview focused on their research agenda, institutional research culture, network, and experience in the Video Showcase. Interviews lasted 45 min to 1 h and were recorded with permission and transcribed for analysis. We also examined the participants’ Video Showcase video presentations and their online discussion posts, which allowed us to triangulate emergent themes. Across the 5 participants, they made an average of 13 posts to their own video and 4 posts to other videos.

### Analysis

**Fig. 1:**
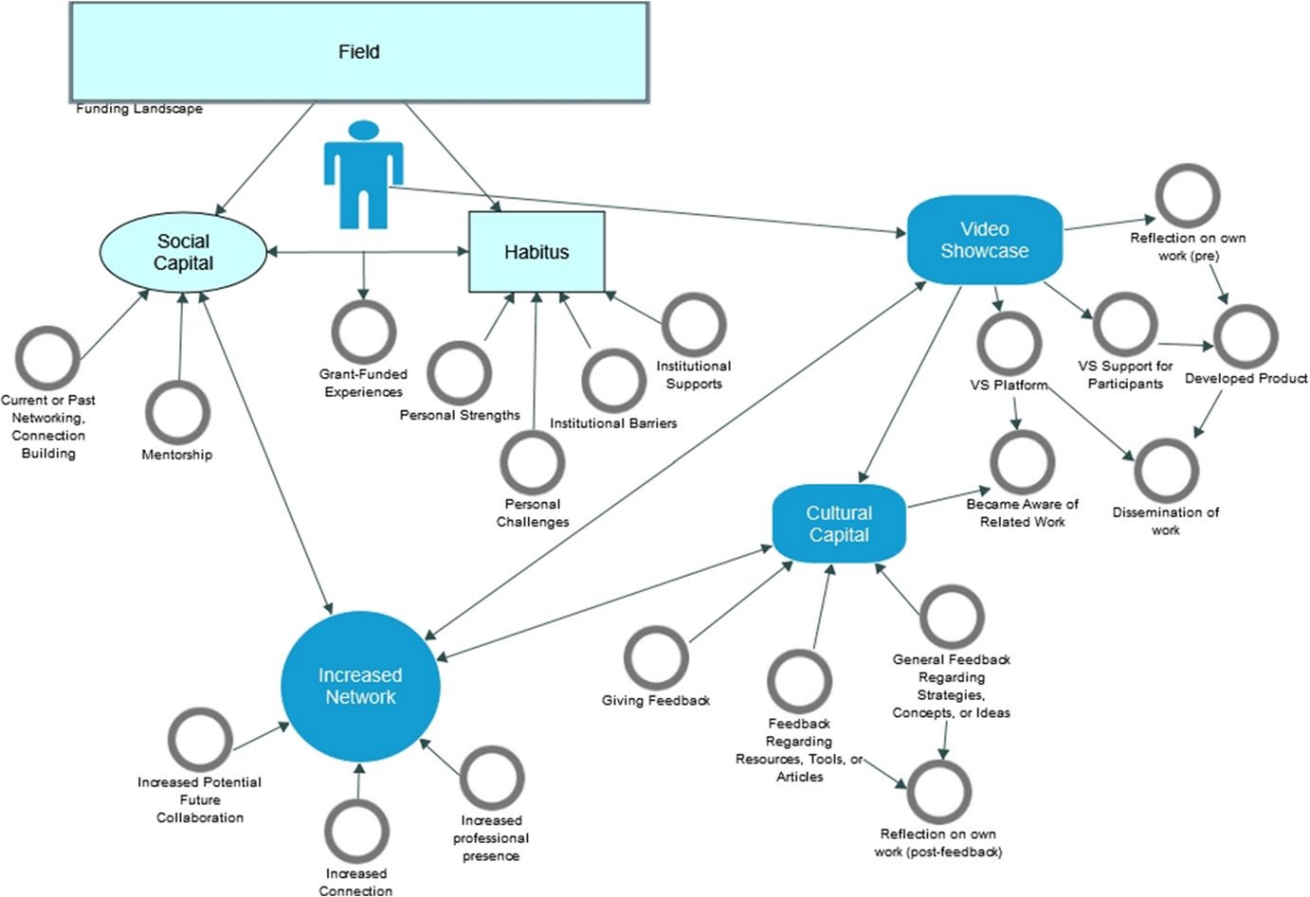
Visualization of codes

During data collection, we wrote memos for each participant, including a profile and initial key words emerging from the interview. The research team reviewed the memos to discuss emergent themes (Maietta, 2006). These memos were used to build inductive codes for the codebook, in addition to deductive codes from the theoretical framework. We used this initial codebook to analyze portions of the interview transcripts, compare our coding to discuss discrepancies, and revise the codebook. We repeated this cycle twice, until our coding was congruent, and we achieved moderate inter-rater reliability. We used the revised codebook to code the interview transcripts in NVivo. Then we used NVivo tools to perform focused coding, which helps researchers examine frequent or significant codes for salient categories in the data (Saldaña, 2013). Examining our analytic memos, case memos, and focused coding, we also developed a network of codes (refer to Fig. 1) to visually organize and display codes, analytic categories, and answer research question (Saldaña, 2013). Data display strategies such as these enhance credibility and trustworthiness of the data analysis (Saldaña, 2013).

## Results

The results are presented in accordance with how the participants entered and experienced the Video Showcase as illustrated in our code visualization (Fig. 1). We found that prior to participating in the Video Showcase, the organizational habitus and field shaped participants’ social capital and grant funded experiences (top left of figure). Participating in the Video Showcase afforded participants an opportunity to develop their research through reflecting on their work, creating a tangible product, becoming aware of related work, and receiving feedback on their work (right side of figure). As an outcome of participating in the Video Showcase, participants were able to expand their network through disseminating the work and increasing their professional presence, as well as increasing their connections and collaborations (bottom of figure). We refer back to the code visualization throughout the findings.

### Organizational habitus and field shapes participants’ social capital and grant funded experiences

Four of the five participants spoke of their institutional cultures valuing research, even though three identified this as a more recent cultural shift. All four mentioned ways their institutions lacked the systems and structures needed to support or advance their capacity for grant funded research. Some participants discussed the lack of structural supports at their institution as related to the institutional field of HBCUs, such as teaching being prioritized over research at HBCUs generally. However, all participants found supports for their research in other ways. In the code visualization (Fig. 1), this finding is represented in the top left by the researcher positioned within a sphere of influence between the field and their organizational habitus and related capital prior to participating the Showcase. For each finding we highlight participant cases as exemplars. Table 2 provides an overview of participant identities.

**Table 2:** Participant profiles

Pseudonym	Race / Ethnicity	Role	Discipline/Field	Institution
Deja Jones	Black	Researcher	Social Science	Public HBCU
Imani Silva	Black	Administrator	Social Science	Public HBCU
Margaret Novak	White	Faculty	STEM	Private HBCU
Ruth Harris	Black	Faculty	STEM	Public HBCU
Tiana Williams	Black	Administrator	Education	Private HBCU

To highlight this finding on organizational habitus and field shaping grant funded experiences and social capital, we offer the case of Ruth Harris.^1^ Dr. Harris was an assistant professor of mathematics at a public HBCU in the Southeastern United States at the time of her interview. Her research focused on the effects of non-cognitive factors on student retention and learning in mathematics, and professional development for K-12 STEM teachers. Dr. Harris was a STEM educational researcher on three National Science Foundation (NSF) funded projects at the time of her interview. She participated in two presentations during the 2021 Video Showcase, one as a co-presenter and one as a lead presenter. She noted that the faculty in her college were able to successfully retain funding for their students. This funding was what kept “the lights on” in her college and provided “opportunities” and the “technology” to support students’ summer research experiences. Overall, she felt research was valued at her institution, even though this was a “pretty new” cultural shift.

While her institution valued and even relied upon research funds, she noted a lack of structural supports. Dr. Harris knew that she could not get course “release time” for research. She noted her “teaching load” could be “scary” when she thought of all her grant funded research time commitments. However, she contextualized her teaching load within the mission of the institution, acknowledging the institution could not offer traditional research development supports (e.g., course release time, reduction of other non-research responsibilities) due to it being a primarily undergraduate teaching university. As Dr. Harris said:

It’s a teaching university, so they can't [give you course release time]… . the support will come more like, ‘Hey this is a great grant, you should really write it. If you need me to read it, I'm willing to read it. [I’ll] help proofread it for you, you can send it to me, and I can help you.’

Despite the lack of structural supports, she often found supports for her research in other ways, such as through her network of internal colleagues. Dr. Harris relied on her department chair for budget questions, and she felt she was supported by “the close-knit” relationships with colleagues in her department, from whom she could seek advice. However, as with all other participants in this study, there was no formal mentoring program at her institution.

Institutional supports and barriers (organizational habitus) shaped researchers’ social capital and grant funded capacity within the field of higher education, with its push for prestige connected to research and call for multi-institution collaborations. Like Dr. Harris, other participants felt their institution valued research, yet found few structural supports to advance their research capacity. Furthermore, all participants mentioned individual people within their institution that they relied upon for advice or support, rather than institutionalized supports that could expand their network beyond their institution and thus their social capital. Other participants mentioned a desire for a “formal mentorship program,” “more infrastructure,” and “clear connections with faculty networks” beyond their institution to avoid “isolation.” Therefore, their organizational habitus, shaped by the field, limited their social capital and restricted their capacity to grow as researchers.

### The video showcase provided participants an opportunity to develop their research

Engaging in the Video Showcase offered participants the opportunity to further develop their research in four interrelated ways: (a) encouraging reflection, (b) developing a product, (c) becoming aware of related work, and (d) receiving feedback. In the code visualization (Fig. 1), this finding is represented in the right-hand side through the “Video Showcase” and “Cultural Capital” nodes. The Video Showcase node includes elements directly resulting from participation (e.g., the reflection that goes into creating a video, developing the video as a product, viewing other videos), while the cultural capital node represents the dispositions of the mind that are valuable within a field (Bourdieu, 1986), such as getting feedback on your work that ideally helps you improve your future work.

#### The video showcase encouraged reflection

In preparation for the Video Showcase, participants had to create a 3-min video highlighting their research. The Video Showcase staff provided participants with online webinars on how to create storylines for an effective video. This was a reflective process that offered participants insight into refining their work as they prepared to present it to a general audience. All five participants discussed the opportunity to reflect on their work through their participation in the Video Showcase.

For example, Imani Silva was program coordinator for an NSF INCLUDES grant at a public HBCU in the Southeastern United States. She was an emerging scholar and was simultaneously pursuing her master’s in a social science field. The members of her team all took active part in making the video, and she thought the process helped them reflect on their progress and future goals. She said:

We spent a lot of time just working together, really figuring out what we want to do. And all of our voices were heard, which I loved… . That really did help us reflect on what we were trying to do and make sure that we get it accomplished.

In this way, the making of the video provided the participants with an opportunity to reflect on their work and come together as a team.

#### The video showcase helped develop a product

After making the video, participants had a product they could use to further disseminate their work and advance future research. Benefits of the video, aside from the presentation itself, included using it as a product in grant renewals, posting it on program websites, and learning video skills to use for future projects. Four of the five researchers discussed ways in which they used the video they developed from their participation in the Video Showcase.

To highlight this point, we offer the case of Deja Jones, a visiting assistant professor and director at a public HBCU in the Southeastern United States. She presented two videos in the 2021 Video Showcase, one on an NSF Established Program to Stimulate Competitive Research (EPSCoR) grant, and the other was for her work as a co-director on an NSF INCLUDES grant. She posted the video on the “larger grant website” and appreciated that it made the site more “interactive” than a typical grant site. Dr. Jones was encouraging her team to create additional videos for “different layers of constituents” because she realized what a “valuable resource” the video medium could be to communicate to different audiences. Further, she was using the Showcase video for “courting partners and supporters” because it was a medium that provided a “good synopsis” of her work without asking people to “read through scores of [written] material.” Therefore, Dr. Jones used the video to disseminate her work to various audiences and advance future, related research.

#### The video showcase increased awareness of related work

The Video Showcase platform also helped all participants become aware of related work, which increased their knowledge related to their research topic. When one is viewing a video, the Showcase webpage presents a list of six related videos, making it easy for presenters to find videos that are similar to theirs, even if funded by different programs within NSF or by a different federal agency. In addition, the Video Showcase allows participants to search all presentations by keyword, age/grade level, funding agency, institutional type, or state to find those of interests. Participants are also able to easily view the videos of researchers who comment on their video through a signature link left in the comment.

An example of the benefit of becoming aware of related work is from the aforementioned case of Ms. Silva. During the Showcase, she used the keyword filter to try to “see what else was out there” related to her video topic on workforce interventions. She became “pretty excited” in “seeing the programs that were a bit similar” to her own. Her goal in watching other researchers’ videos was to “see the variety of what’s out there and [what] other people are doing … how we can try and use that.” The multimodal nature of the Showcase afforded her the opportunity to explore similar programs through the video medium. Becoming aware of others who are doing similar work was important for Ms. Silva, as an important part of her hub program was to build relationships with nearby workforce programs. As an early career scholar, she was also looking to expand her professional presence and build connections.

#### Receiving feedback on work from the video showcase

The online discussion forum appended to each video on the Showcase offered feedback to the presenters. All participants mentioned some type of feedback from their video’s discussion, whether it was critiques of their work, ideas, or resources. This feedback helped researchers reflect on their work moving forward, as well as build contacts for future work as discussed in the next section.

For example, Dr. Jones, previously discussed, was an established scholar and had received other research grants from multiple federal agencies. She entered the Video Showcase hoping to address specific challenges she was facing in her program, as evidenced by her video’s introductory discussion post asking for “some feedback on the following: [the intervention framework’s] transferability to higher education [and] suggestions for sustaining faculty support and training beyond the grant.” Dr. Jones noted their video received quality feedback, where “people brought a different type of theoretical model, that may be an additional explanation of what we were seeing, which was good.” This feedback on their video, which also increased their awareness of related work, sparked a conversation between the research team and one commenter. The subsequent conversations helped Dr. Jones “flesh out” and “refine” their theoretical model, which they are now using for “another grant proposal.” Dr. Jones and her team “go back to those videos’ [discussion forums] now to look at the input … to have some continued conversation and development in the team” as they refine their work. Therefore, as the online discussion forums connected to each video are archived, they serve as a stable resource to return to and to explore theories and ideas to further inform their work.

### The video showcase provided participants an opportunity to expand their network

The Video Showcase provided participants an opportunity to expand their professional network as well. We have conceptualized an enlarged network as comprising two categories: (a) increased professional presence and dissemination of work, and (b) increased number of connections with potential future collaborators. All participants found the Video Showcase offered them a way to disseminate their work and enhance their professional presence, and increase connections in their network. Four of the five discussed connecting with potential future collaborators. In the code visualization (Fig. 1), this finding is represented in the bottom left, indicating how an increased network was related to both participation in the Video Showcase as well as their prior social capital (their network and mentorship before entering the Showcase).

#### The video showcase disseminated work and enhanced professional presence

To illustrate this finding, we draw on the case of Tiana Williams. Ms. Williams was an administrator and adjunct professor at a private HBCU in the Southeastern United States, and the lead presenter of a 2021 Video Showcase presentation on her NSF HBCU Undergraduate Program (HBCU-UP) grant. As an early career scholar and doctoral student, she desired to disseminate her work to inform practice. At the encouragement of her mentor, she participated in the Video Showcase. Afterwards, her mentor showed her the activity map which the Showcase provides for each presenter, displaying viewers’ geolocations. She realized that people as far as “Hong Kong” had watched her video. While they were both “excited” that she was expanding her professional presence, it was also about increasing the reach of her project. Ms. Williams said:

What [my mentor] eventually taught me was this is about a claim. This is about you getting your work out there to the masses. At first, I didn’t look at it from that lens. I looked at it from the point of view of I’m helping these students [engaged in the research program], wow. This is a great thing. I didn’t look at it as far as, no, you’re not only helping these students in this small pocket of the world, you eventually can touch just about everybody. So, that was very eye-opening as well.

Like Ms. Williams, other participants used the Video Showcase to disseminate their work by promoting the link to their presentation through email and social media, or by embedding their video on other websites.

#### The video showcase increased connections and collaborations

Margaret Novak, an assistant professor of biology and environmental science at a private HBCU in the Southeastern United States, provides an example of finding increased connections and potential future collaborators through the Showcase. She was the lead presenter of a 2020 Video Showcase presentation on an NSF HBCU-UP grant, and a co-presenter of a 2021 presentation on a Division of Ocean Sciences NSF grant. During the 2020 Showcase, she had a higher-than-average number of posts on her video (36) and she viewed and commented on nine other videos, engaging other researchers with related work. After her 2020 video, she was invited to participate in a STEM for All Multiplex theme of the month webinar panel (which brought together several presentations related to a similar theme from the Video Showcase) where her research was highlighted. Her participation on that panel increased her professional presence and attracted the attention of another research group at a different university. She said, “they had seen the video that I made [for the Video Showcase] and then saw me in the [Multiplex] webinar” and “reached out” to “collaborate” on a grant. This project resulted in her second Video Showcase presentation in 2021. Dr. Novak felt there were multiple instances in both Video Showcase discussions where “there was a lot of potential for more collaborations.” As other study participants commented, some of the connections made during the Showcase resulted in collaborative work, and some remained at the connection level but still expanded their professional network.

## Discussion and implications

Results indicate that prior to the Video Showcase, organizational habitus and field shaped participants’ social capital and grant funded experiences. The Showcase offered participants an additional opportunity to develop their research and expand their network in tangible ways. These findings add to the literature by indicating how a cross funded program, cross university, free online platform could help researchers whose organizational habitus limits their research capacity build their networks and social capital. We situate these findings in the literature, and offer recommendations for practice, policy, and future research.

### Organizational habitus and field

Participants said that their institutional cultures valued research, but that they lacked the systems and structures to support or advance their capacity for grant funded research. This finding aligns with the literature on supports and barriers at HBCUs and other undergraduate teaching institutions (Ellis, 2012; Pinto & Huizinga, 2018). Institutional factors are highly predictive of researchers’ grant-seeking and research productivity. For example, Pinto and Huizinga (2018) found that a high teaching load and an increasing demand for grant funded research, yet no centralized support office, lead to decreased research capacity for researchers at an MSI. This finding reflects the organizational habitus experienced by the participants in this study as well. Some participants in Ellis’s (2012) study on the socialization and retention of Black junior faculty felt it was “academic suicide” to take an appointment at an HBCU because of the institutional demands on their time and lack of adequate research support (p. 145). Studies have found that the lack of state and federal funding at HBCUs (Boland & Gasman, 2014; Pece, 2018, 2019; Sav, 2010) can result in limited resources such as sponsored programs office staff (Matthews & Coleman, 2011) and laboratory space (Qazi & Escobar, 2019). Like the participants in this study, Ellis’s (2012) participants also relied on individuals in their circle for guidance in advancing their careers. Yet the importance of research at MSIs and other teaching institutions is increasing, and that pressure is passed on to researchers without the structures in place to support this mission shift (Gonzales, 2013; Johnson & Harvey, 2002).

Based on this finding, HBCUs and other teaching institutions can identify ways to institutionalize support for grant funded research in ways that do not cost additional resources. For example, HBCU sponsored programs staff could partner with other institutions or federal programs to offer workshops and resources beyond the introductory level professional development typically offered (Toldson, 2017). Institutions may also adjust their policies for tenure and promotion. For example, Fields (2020) suggests protecting the time of early career scholars as they get acclimated to their roles, particularly Black women, as they are often assigned an inequitable amount of service. Lastly, if an institution cannot protect faculty time due to teaching and service requirements as part of their mission, administrators may need to examine tenure policies to make sure they align with institutional priorities.

### Multimodal professional network providing opportunities to develop research

We found that the Video Showcase provided participants with a valuable opportunity to develop their research through reflection, to increase their knowledge of related work, and to meet other researchers in their field and receive feedback from them. While a multimodal professional network like the Video Showcase does not have the ability to change the field of higher education, alter the organizational habitus of HBCUs, or create more equity between institutional types, it is an intervention that increases the capacity of individual researchers for grant funded research. The advantage of this intervention is accelerated through the multimodal nature of the environment, which includes video sharing, collegial discourse, and creating a feedback loop for developing research. For example, participants noted the value of the reflection process in creating a video, which was then used to disseminate their work and to further establish their professional identity. In turn their video presentation and related discussion elicited feedback from others which contributed to improvement of current work, as well as finding potential collaborators for future work—expanding their social capital. The fact that this happened asynchronously online, gave presenters both a developed product that could be reused and archived feedback for later retrieval (Falk et al., 2019).

There is little research on feedback and reflection as part of professional conferences broadly, and multimodal professional network environments specifically. Some scholars have examined the nature of poster presentation modality, including how design fosters reflection and promotes knowledge transfer (Ilic & Rowe, 2013), and how the casual interactional nature of posters can provoke constructive criticism for improving future work (Garvey et al., 1972; Lengalova, 2016). However, these poster presentations happen synchronously and therefore lack key benefits of the Video Showcase’s multimodal environment for sustained and archived collegial discourse.

An implication of this finding is the need for further exploration of the benefits of multimodal professional network environments for researchers’ professional development. As more journals move from print to online and offer multimodal content, and conferences move toward hybrid and online formats due to COVID-19, we need to further understand the influence of these innovations on researchers and their work. This trend will only likely increase as it offers accessibility and engagement beyond a place-based and synchronous format, opening this intervention to international researchers and audiences. Further, as federal agencies prioritize broadening participation in STEM, they have begun to encourage researchers to diversify their scientific communication to reach broader, nonacademic audiences (Farmer Cox et al., 2014). Better understanding of the nature, benefits, and limitations of such professional networks may open the way to improved designs for this purpose. This study just begins to touch on these themes, as it explored the impact of a multimodal professional network environment for a specific population—HBCU-based researchers.

### Multimodal professional network expanding professional networks

The Video Showcase provided study participants with an opportunity to expand their network in tangible ways, which enhanced their social capital. Research draws a positive relationship between collaboration and scholarly productivity (Baldwin & Austin, 1995; Bankart, 2012; Hitchcock et al., 1995), including the number of peer-reviewed journal articles (Lee & Bozeman, 2005). Furthermore, funders are calling for multi-institution and collaborative proposal submissions (Dopke & Crawley, 2013; Eddy, 2010; Lee & Bozeman, 2005). Collaborations with researchers at other institutions is also a strategy for MSI-based researchers to maintain an active research agenda if their home institution lacks centralized sponsored program support (Pinto & Huizinga, 2018).

 A myriad of research findings points to the importance of mentorship as related to collaboration and scholarly productivity, especially for Black faculty (e.g., Ellis, 2012). However, collaboration can be lacking in HBCU research culture (Qazi & Escobar, 2019), and it can be difficult for Black faculty to find trusted mentors as they are underrepresented in the field (Baldwin & Griffin, 2014). We found that those who mentioned strong mentor guidance, which perhaps indicates a stronger social network prior to entering the Showcase, left the Video Showcase with more connections and future potential collaborators. This speaks to Bourdieu’s notion of social capital as a mechanism of social reproduction. Perhaps you need some initial social capital to begin with to expand your network further. Implications of this finding suggest that HBUCs could enhance collaboration through: (a) mentorship and internal relationships, (b) tenure and promotion policies, and (c) external relationships and networks.

First, all study participants noted that their institutions had no formal mentoring program. Formal mentoring programs often provide structure and guidance for early career scholars that is needed for scholarly success and productivity, particularly for Black women faculty (Fields, 2020; Kelch-Oliver et al., 2013). Moreover, to further develop internal relationships and connections, Kelly and Winkle-Wagner (2017) suggest deans could create cross-departmental support networks, luncheons, or colloquiums that bring people together across departments to know and support one another.

Second, tenure and promotion policies could be adjusted to facilitate collaboration. Baldwin and Austin (1995) found a common pattern of “institutional ambivalence toward collaboration and the absence of clear guidelines” (p. 64). With no formula for “gauging the merit of collaborative publications” it can be unclear for faculty how single- or collaborative-authored papers count toward tenure (p. 64). This may discourage junior faculty from collaborating. Institutions may also offer incentives and rewards outside of tenure policies that encourage collaboration (Baldwin & Austin, 1995).

Finally, HBCUs could encourage researchers to develop external relationships and networks. For example, Toldson (2017) found that a driver of success in HBCU-based researchers obtaining external funding was developing relationships with organizations, foundations, and government agencies that provide funding. This can aid researchers in seeking funding and proposal preparation. Other scholars provide examples of networks that offer research development funded by external agencies. For example, Wheaton and Moore (2019) explain how they created a network of STEM scholars across MSIs to advance the scholarly productivity of racially minoritized women. Pettit (2019) also provides an example of a consortium of multiple colleges that offer an annual summer workshop on faculty socialization for racially minoritized faculty. Strategic partnerships and collaborations outside of one’s institution can advance the social capital and research capacity of HBCU-based researchers.

## Limitations

The aim of interpretive qualitative research is not to generalize, but rather through analysis arrive at “concrete universals”—that is, findings that could be similar across cases (Erickson, 1986, p. 130). The sample for this study is limited to a particular context (i.e., the individual researchers and their specific institutions), and lacked diversity on some dimensions (e.g., having no men as participants). However, the strength of a case study is that it offers detailed examples and context-dependent knowledge (Mertens, 2009).

## Conclusion

In this study, we set out to examine how a multimodal professional network created to allow faculty to share and discuss their research, affected HBCU-based researchers’ capacity to engage in grant funded research. We found the organizational habitus of HBCUs and the field of federal funding in higher education shaped participants’ social capital and grant funded research experiences prior to participating in the Video Showcase. The HBCU-based researchers found that participating in the Video Showcase provided an opportunity to further develop their research and expand their professional network in tangible ways. These findings illustrate how this multimodal professional network environment increased the dissemination of researchers’ work, enhanced their professional presence in the field, enabled them to receive ideas, feedback, and resources that would further their current research efforts, and finally, make connections for future collaborations on future grant proposals.

While we noted that multimodal professional networks like the Video Showcase would not necessarily change the field of higher education, the creation of more multimodal professional networking environments aimed at fostering and enabling collaboration, could help bridge the gap of inequity between organizational types. Federal agencies who award these grants could consider funding more multimodal professional networks, such as the NSF has done for the STEM for All Video Showcase. These networks build capacity across agencies and institutional types, enhance research currently funded, and propel new collaborative efforts in the future. Many agencies are interesting in funding HBCU-based researchers yet struggle to do so. Therefore, multimodal professional networks are a powerful way to enhance HBCU-based researcher capacity to apply for and receive federal funding.

## Data Availability

Some datasets used and/or analyzed during the current study are available from the corresponding author upon reasonable request, and other datasets are publicly available on the STEM for All Multiplex website (https://multiplex.videohall.com/).

## Footnotes

1 All names are pseudonyms.
